# Magnitude and determinants of intimate partner violence against women in East Africa: multilevel analysis of recent demographic and health survey

**DOI:** 10.1186/s12905-022-01656-7

**Published:** 2022-03-17

**Authors:** Sewnet Adem Kebede, Adisu Birhanu Weldesenbet, Biruk Shalmeno Tusa

**Affiliations:** 1 Department of Epidemiology and Biostatistics, Institute of Public Health, College of Medicine and Health Sciences, University of Gondar, Gondar, Ethiopia; 2 Department of Epidemiology and Biostatistics, College of Health and Medical Sciences, Haramaya University, Haramaya, Ethiopia

**Keywords:** Intimate partner violence, East Africa, Multilevel analysis

## Abstract

**Background:**

Violence against women is a significant public health problem, and human rights abuse, and is associated with multiple adverse physical, mental, sexual, and reproductive health effects. The current study aimed to determine the magnitude of intimate partner violence (IPV) and its determinant factors in East African countries.

**Methods:**

We utilized the most recent demographic and health survey data from 11 East African countries, which was comprised of a weighted sample of 55,501 ever-married women. A multilevel multivariable logistic regression analysis was applied. We used an adjusted odds ratio with a 95% CI and a *p* value ≤ 0.05 in the multilevel logistic model to declare significant factors associated with IPV.

**Results:**

The overall prevalence of all forms of IPV in East African countries was 32.66% [95% CI 32.27, 33.05], with the highest IPV occurring in Uganda (14.93%) and the lowest IPV recorded in Comoros (0.87%). In the multivariable multilevel logistic regression model, women’s education, residence, sex of household head, current pregnancy, husband drinking alcohol, attitude towards wife-beating husband controlling behavior, and women’s decision-making autonomy were significantly associated with IPV.

**Conclusion:**

The risk factors noted above increase the likelihood of a woman experiencing IPV. Therefore, we recommend establishing effective health and legal response services for IPV, raising awareness of the existing legislation service and improving its application, strengthening legislations on purchasing and selling of alcohol, strengthening joint (both husband and wife) decision-making power by empowering women, improving the educational level of women, and establishing measures to break the culture of societal tolerance towards IPV.

**Supplementary Information:**

The online version contains supplementary material available at 10.1186/s12905-022-01656-7.

## Background

Intimate partner violence (IPV) refers to any behavior within an intimate relationship that causes physical, psychological, or sexual harm to those in the relationship [1]. It is one of the most prevalent types of violence against women, and it involves physical violence such as slapping, striking, kicking, and beating, as well as sexual violence, mental abuse, and controlling behaviors by an intimate partner [2, 3]. Violence against women is a significant public health problem, a human rights abuse, and is associated with multiple adverse physical, mental, sexual, and reproductive health effects [4].

Globally, 30% of women have experienced physical and/or sexual violence by their intimate partners in their lifetime [5]. According to studies, 13–61% of IPV victims have experienced physical violence by a partner, 4–49% have experienced severe physical violence by a partner, 6–59% have experienced sexual violence by a partner at some point in their lives, and 20–75% have experienced one emotionally abusive act in their lifetime. The percentage of ever-partnered women who reported ever experiencing any physical or sexual violence by their current or most recent husband or cohabiting partner ranged from 18% in Cambodia to 48% in Zambia for physical violence, and 4% to 17% for sexual violence. According to a study from 10 countries, physical or sexual IPV ever reported by currently married women ranged from 17% in the Dominican Republic to 75% in Bangladesh [6–8].

In low- and middle-income countries, in which most African countries are included, the prevalence of IPV is much higher due to the high social acceptance of violence and poor socioeconomic status, with studies reporting 36% of violence during pregnancy. In Africa, IPV during pregnancy ranged from 2.3 to 57.1% [9, 10]. Low levels of education and a lack of decision-making power in these regions make women more dependent on their male partners and increase their likelihood of experiencing violence [10].

Intimate partner violence has both short-term and long-term effects on women’s physical and mental health, and its adverse effects sometimes extend to infants of women who experienced IPV. History of experiencing violence is therefore a risk factor for many diseases and conditions [3]. Alcohol and drug abuse, eating and sleep disorders, physical inactivity, poor self-esteem, post-traumatic stress disorder, smoking, and self-harm are major consequences of IPV among women, and children, anxiety, depression, poor school performance, and unfavorable health outcomes are major threats [11].

Even though the prevalence of IPV has remained significant worldwide, no study has been conducted to investigate the extent and related determinants of IPV against women in Eastern Africa. As a result, the current study used Demographic and Health Survey (DHS) data to determine the magnitude of IPV and its determinant factors in East Africa. The finding of this study provides evidence for planners, decision-makers, stakeholders, and health professionals in planning for the reduction of IPV, which is helpful to overcome the negative consequences of IPV.

## Method

### Study period and setting

We conducted secondary data analysis using the most recent demographic and health survey (DHS) in the following East African countries (Burundi, Ethiopia, Kenya, Comoros, Malawi, Mozambique, Rwanda, Tanzania, Zambia, Zimbabwe, and Uganda) from 2012 to 2018.

### Data source

The data were accessed from the demographic and health survey (DHS) program official database www.measuredhs.com. Demographic and health surveys are nationally representative household surveys that provide data that is comparable across the countries for monitoring and impact evaluation indicators in the areas of population, health, and nutrition. The DHS uses a stratified two-stage cluster design: enumeration areas (EA) (first stage) and in each EA selected, a sample of households is drawn (second stage). Detailed survey procedure [12].

For this study, individual record (IR) datasets were used in each country. The data were obtained from all ever-married women 15–49 selected and interviewed for the violence module in each country. We used DHS surveys done in 11 East African countries and a weighted sample of 55,501 ever-married women from Burundi (6558), Ethiopia (4469), Kenya (4023), Comoros (2093), Malawi (4984), Mozambique (5610), Rwanda (1691), Tanzania (7102), Uganda (6879), Zambia (6598) and Zimbabwe (5494) were included in the study.

### Measurements of variable and operational definitions

#### Outcome variable

The outcome variable for this study was IPV. Intimate partner violence is defined as any behavior within an intimate relationship that causes physical, emotional, or sexual harm to those in the relationship, whether current or former spouses or current or former partners [1] (Additional file 1).

##### Physical violence

Ever-married women who have experienced one or more of the specified acts (any of pushing you, shaking you, or throwing something at you; slapping you; twisting your arm or pulling your hair; punching you with his/her fist or with something that could hurt you; kicking you, dragging you, or beating you up; trying to choke you or burn you on purpose; or threatening or attacking you with a knife, gun, or any other weapon) [1].

##### Sexual violence

Ever-married women who have experienced one or more of the specified acts (any physical force you to have sexual intercourse with him even when you do not want to; physically forcing you to perform any other sexual acts you do not want to; forcing you with threats or in any other way to perform sexual acts you do not want to) [1].

##### Emotional violence

Ever-married women who have experienced one or more of the specified acts (any of saying or doing something to humiliate you in front of others; threaten to hurt or harm you or someone close to you; insult you or make you feel bad about yourself) [1].

##### Intimate partner violence

Ever-married women who have experienced physical violence or sexual violence or emotional violence by their current or most recent husbands/partners in the 12 months preceding the survey.

#### Explanatory variable

##### Community media exposure

It was defined as the proportion of women who had media exposure in a cluster. The aggregate of individual women’s media exposure can show the overall media exposure of women within the cluster. It was categorized into higher and lower community media exposure based on national median value since these were not normally distributed. *Community Paternal education* was defined as the proportion of husbands who attended primary, secondary, and higher education within the cluster. The aggregate of an individual husband’s primary, secondary, and higher educational attainment can show the overall educational status of the husband within the cluster. They were categorized into two categories: those with a higher proportion of the husband’s education within the cluster and those with a lower proportion of the husband’s education based on the national median value since these were not normally distributed.

##### Attitude towards wife-beating

It was measured based on the following five questions that ever-married women were asked about whether situations of hitting or beating a wife were justifiable in the following situations: if she goes out without telling him; neglects their children; argues with him; refuses to have sex with him, and burns the food. If they said "yes" to any one of the above questions, they were classified as having an attitude towards wife-beating [13].

##### Husband controlling behavior

Five questions were used in this study to assess husband controlling behavior. Ever married woman was asked about her husband controlling behavior. Husband jealous if respondent talks with other men, husband accuses respondents of unfaithfulness, the husband doesn’t permit respondent to meet female friends, husband insists on knowing where the respondent is or husband tries to limit respondent’s contact with family [14].

### Data management and analysis

After extracting the variables based on literature, we pooled the data from 11 East African countries together. Before any statistical analysis, the data were weighted using sampling weight, primary sampling unit, and strata to compensate for under or over-representing certain households in a sample, allowing it to reflect the population as a whole. Because of clustering and sampling, virtually all random sample surveys must use weights to make estimates that are valid for the whole population. The pooled prevalence of IPV with a 95% confidence interval (CI) was reported for East African countries from 2012 to 2018.

Multilevel multivariable logistic regression analysis was used since the outcome variable is binary (“1” if women experience IPV and “0” otherwise). We fitted four models to identify determinant factors of IPV. The first model is the null model without determinant factor, the second model is the model with only individual-level factors, the third model is the model with only community-level factors, and the final model with both the individual and community-level factors. Variables with a *p* value < 0.2 in the bivariable analysis were considered in the multivariable multilevel logistic regression model. We used an adjusted odds ratio (AOR) with a 95% CI and *p* value ≤ 0.05 in the multilevel logistic model to declare significant factors associated with IPV.

## Result

### Socio-demographic characteristics

A total of 55,501 ever-married women aged 15–49 from 11 East African countries were included in this study. The median age of the women was 30 years old, with an interquartile range of 25 to 37, and about 38% of women were aged 20–29 years. Most women lived in rural areas (72.53%) and only 4% of women attended higher education. More than half of the study participants were working at the time of the survey (Table 1).

**Table 1:** Individual and community-level characteristics of IPV in East African countries according to recent demographic and health surveys from 2012 to 2018

Variables	Weighted frequency	Percentage (%)
Women age 36 ± 11		
15–19	3349	6.03
20–24	9750	17.57
25–29	11,268	20.30
30–34	10,493	18.91
35–39	8846	15.94
40–44	6721	12.11
45–49	5074	9.14
Place of residence		
Urban	15,244	27.47
Rural	40,257	72.53
Women educational level		
No education	13,233	23.84
Primary	27,615	49.76
Secondary	12,381	22.31
Higher	2272	4.09
Sex of household head		
Male	39,996	72.06
Female	15,505	27.94
Country		
Burundi	6558	11.82
Ethiopia	4469	8.05
Kenya	4023	7.25
Comoros	2093	3.77
Malawi	4984	8.98
Mozambique	5610	10.11
Rwanda	1691	3.05
Tanzania	7102	12.80
Uganda	6879	12.39
Zambia	6598	11.89
Zimbabwe	5494	9.90
Wealth index		
Poorest	11,091	19.98
Poorer	10,949	19.73
Middle	10,797	19.45
Richer	11,192	20.17
Richest	11,472	20.67
Partner education (48,515)		
No education	8512	17.54
Primary	22,493	46.36
Secondary	12,995	26.79
Higher	3466	7.14
Don’t know	1049	2.16
Women currently working		
No	19,639	35.39
Yes	35,862	64.61
Media exposure		
No	18,194	32.78
Yes	37,307	67.22

### Magnitude of intimate partner violence

**Fig. 1:**
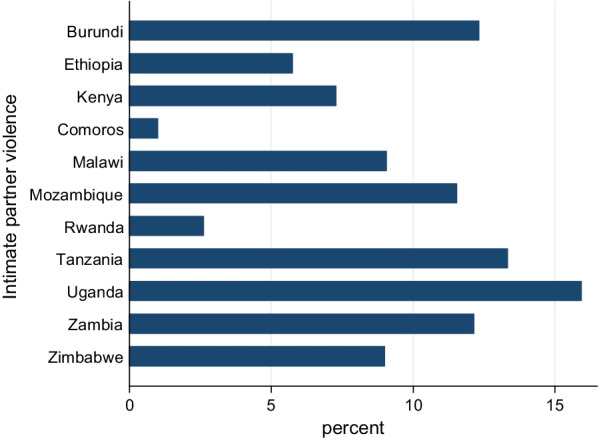
Magnitude of intimate partner violence in East African countries recent demographic and health surveys from 2012 to 2018

The overall magnitude of all forms of IPV in East African countries was 32.66% [95% CI 32.27, 33.05], with the highest IPV occurring in Uganda (14.93%) and the lowest IPV recorded in Comoros (0.87%) (Fig. 1).

### Determinants of intimate partner violence

Intra-cluster Correlation Coefficient (ICC) and Likelihood ratio (LR) tests were checked. The best-fitted model for the data was a two-level multilevel logistic regression model. The ICC in the null model was 0.15 (95% CI 0.14, 0.16), indicating that the variations between clusters were responsible for around 15% of the overall variability of IPV and the remaining was attributable to individual differences (Table 2).

**Table 2:** Multivariable multilevel logistic regression model results of IPV in East African countries recent demographic and health surveys from 2012 to 2018

Variable	Null model	Model I (AOR, 95% CI)	Model II (AOR, 95% CI)	Model III (AOR, 95% CI)
Residence				
Urban			1	1
Rural			1.22 (1.12, 1.31)	1.16 (1.14, 1.47)
Community media exposure				
Lower			1	1
Higher			0.99 (0.93, 1.06)	1.13 (0.89, 1.44)
Community paternal education				
Lower			1	1
Higher			1.11 (1.04, 1.20)	0.95 (0.75, 1.21)
Woman’s age				
15–19		1		1
20–24		0.97 (0.74, 1.28)		0.98 (0.75, 1.29)
25–29		0.98 (0.72, 1.35)		0.99 (0.73, 1.36)
30–34		0.82 (0.56, 1.18)		0.82 (0.56, 1.19)
35–39		0.69 (0.44, 1.11)		0.70 (0.44, 1.12)
40–44		0.89 (0.47, 1.69)		0.91 (0.49, 1.72)
45–49		1.13 (0.41, 3.04)		1.09 (0.40, 2.93)
Women’s education				
No education		1		1
Primary		0.88 (0.71, 1.01)		0.83 (0.66, 1.05)
Secondary		0.74 (0.55, 1.01)		0.68 (0.49, 0.94)
Higher		0.76 (0.40, 1.44)		0.69 (0.36, 1.32)
Sex of household head				
Male		1		1
Female		0.77 (0.61, 0.96)		0.77 (0.61, 0.97)
Age of household head		1.01 (0.99, 1.02)		1.01 (0.99, 1.02)
Wealth index				
Poorest		1		1
Poorer		0.88 (0.70, 1.12)		0.88 (0.70, 1.12)
Middle		1.15 (0.90, 1.47)		1.15 (0.90, 1.47)
Richer		0.94 (0.72, 1.22)		0.94 (0.72, 1.24)
Richest		0.71 (0.51, 0.97)		0.71 (0.50, 1.01)
Number of children		1.03 (0.96, 1.09)		1.02 (0.96, 1.09)
Current pregnancy wanted				
Then		1		1
Later		1.24 (1.04, 1.49)		1.24 (1.04, 1.49)
Not at all		1.36 (0.98, 1.89)		1.37 (1.01, 1.91)
Paternal education				
No education		1		1
Primary		1.13 (0.89, 1.45)		1.11 (0.87, 1.41)
Secondary		0.95 (0.71, 1.28)		0.92 (0.68, 1.25)
Higher		0.84 (0.48, 1.46)		0.81 (0.46, 1.41)
Don’t know		0.85 (0.47, 1.54)		0.81 (0.45, 1.48)
Husband age		0.99 (0.98, 1.01)		0.99 (0.98, 1.01)
Husband drinks alcohol				
No		1		1
Yes		3.45 (2.90, 4.10)		3.45 (2.91, 4.11)
Attitude towards wife beat				
No		1		1
Yes		1.70 (1.44, 2.01)		1.71 (1.45, 2.02)
Husband controlling behavior				
No		1		1
Yes		6.31 (5.18, 7.67)		6.21 (5.09, 7.56)
Women’s decision-making autonomy on				
Her health care				
No		1		1
Yes		0.82 (0.67, 1.00)		0.82 (0.66, 0.99)
Major household purchases				
No		1		1
Yes		0.78 (0.64, 0.95)		0.78 (0.64, 0.95)
Visits to her family				
No		1		1
Yes		1.36 (1.11,1.67)		1.37 (1.12,1.68)
Model comparison and random effect				
ICC	0.15 (0.14, 0.16)			
Log-likelihood	− 33,873.6	− 29,241.1	− 33,831.6	− 29,216.4
Deviance	67,747.2	58,482.2	67,663.2	58,432.8

AOR, adjusted odds ratio; CI, confidence interval; ICC, intra-class correlation coefficient

In the multivariable multilevel logistic regression model, women’s education, residence, sex of household head, current pregnancy, husband drinking alcohol, attitude towards wife beat, husband controlling behavior, and women’s decision-making autonomy were significantly associated with IPV.

Living in the rural parts of East Africa increased the likelihood of experiencing IPV by 16% (AOR = 1.16, 95% CI 1.14, 1.47) as compared with living in the urban areas of East Africa. The likelihood of experiencing IPV was decreased by 32% among women who had a secondary level education as compared to women who had no education (AOR = 0.68, 95% CI 0.49, 0.94).

The odds of experiencing IPV were decreased by 23% among female-headed households as compared to male-headed households (AOR = 0.77, 95% CI 0.61, 0.97). Intimate partner violence was more likely to occur among women who had an unintended pregnancy (wanted later pregnancy and/or unwanted pregnancy) (AOR = 1.24, 95% CI 1.04, 1.49) and (AOR = 1.37, 95% CI 1.01, 1.91) than women who had wanted pregnancy.

A husband who drinks alcohol was 3.45 times more likely to commit physical, emotional, or sexual violence on his wife as compared to a husband who didn’t drink alcohol (AOR = 3.45, 95% CI 2.91, 4.11). The odds of IPV among women who had an attitude toward wife beating were increased by 71% as compared to women who had no attitude toward wife-beating (AOR = 1.71, 95% CI 1.45, 2.02). Husbands who had controlling behavior were 6.21 times more likely to commit physical, emotional, or sexual violence on their wives as compared to a husband who had no controlling behavior (AOR = 6.21, 95% CI 5.09, 7.56).

The probability of IPV among women who had decision-making autonomy on their health care and major household purchases decreased by 18% (AOR = 0.82, 95% CI 0.66, 0.99) and 22% (AOR = 0.78, 95% CI 0.64, 0.95) than their counterpart respectively. Women who had decision-making autonomy to visit their families increased the occurrence of IPV by 37% as compared to their counterparts (AOR = 1.37, 95% CI 1.12, 1.68).

## Discussion

Intimate partner violence affects millions of women of all ages globally. It has both direct and indirect lifelong impacts on their health. Therefore, knowing the magnitude and associated factors of IPV in East Africa may offer evidence for East African countries' policymakers to design targeted prevention and intervention programs aimed at decreasing IPV and preventing risk factors. The pooled prevalence of IPV in East African countries was 32.66% [95% CI 32.27, 33.05], with the highest IPV occurring in Uganda (14.93%) and the lowest IPV recorded in Comoros (0.87%). This was consistent with the studies done in Ivory Coast [15, 16]. The finding was lower than the study conducted in western African countries [17, 18].

In the multivariable multilevel logistic regression model, women’s education, residence, sex of household head, current pregnancy, husband drinking alcohol, attitude towards wife beat, husband controlling behavior, and women’s decision-making autonomy were significantly associated with IPV in East African countries.

The study showed that the residence of the respondents had a significant association with IPV. Women who were from a rural part of East African countries were found to experience IPV as compared with those from the urban part of East African countries. This finding is in line with other studies done in Iran and Ethiopia [19, 20]. Women living in rural areas experience a higher rate of IPV. This could be explained by women who do seek help but find difficulty in accessing services due to geographical isolation, lack of transportation, and not having access to their income [21, 22]. Additionally, rural parts of the countries had no or few institutions that help to intervene against IPV or prevent the violence before its occurrence. There may be also cultural value differences or disparity.

In the current analysis, educated women were less likely to experience IPV as compared to women who had no formal education, which has been confirmed in previous studies [23, 24]. The possible explanation could be education is one of the mechanisms to empower and develop a sense of self-esteem among women.

This study showed that IPV occurrence was decreased among female-headed households as compared to male-headed households, which is in contrast with the study done in India [25]. This discrepancy could be due to cultural differences.

Consistent with the previous studies [26–28], unintended pregnancy was found to have a significant association with IPV. This could be explained by women who experienced IPV and unintended pregnancy mostly living together in situations without a good relationship. In another direction, unintended pregnancy may be occurring due to sexual violence by a partner or husband.

Intimate partner violence increased linearly with the husband’s alcohol intake. This finding is consistent with the study which was done in Gambia, Ethiopia, Ghana, and Malawi [17, 18, 28, 29]. The possible explanation could be the effect of alcohol on the cognitive capabilities, reducing self-control of individuals lower inhibitions, and heightening patriarchal ideologies, thus arousing dominant toxic masculinities. After drinking alcohol, the user may behave aggressively and leave individuals less capable of negotiating a non-violent resolution to the problem within the relationship. Furthermore, excessive drinking can exacerbate financial difficulties, children’s problems, or other family stressors. This can create marital tension and conflict, increasing the risk of violence [30].

The present study documented those women who have an attitude towards wife-beating were more likely to experience IPV. This finding is consistent with the studies done in Zimbabwe [31]. The possible explanation could be over time they may develop tolerant attitudes toward IPV violence against women and consider the violence as normal in their life process.

A direct strong relationship exists between husband controlling behavior and occurrence of IPV against women. This finding is most consistent with the findings of previous studies in Ghana [17] and Gambia [18]. This could be explained by community perception of male superiority which is expressed through control of women.

The odds of IPV among women who had decision-making autonomy on their health care and major household purchases decreased by 18% and 22% than their counterparts respectively. This finding is in line with other reports elsewhere [31–33]. While women who had decision-making autonomy to visit their family was negatively associated with IPV. Women who had decision-making autonomy to visit their family increase the occurrence of IPV by 37% as compared to their counterparts which are in line with the study done in Sub-Saharan Africa [23]. The possible explanation could be that women who have decision-making autonomy are able to fight for their rights and resist some of the decisions of men.

### Strength and limitation

The data used in this study was representative of 11 East African countries and we must consider heterogeneity. In this study, some limitations were presented. Firstly, the findings may not build a causality relationship between participant characteristics and IPV experience due to the cross-sectional nature of the study. Secondly, underreporting of IPV due to social desirability bias.

## Conclusion

In East Africa, near to one-third of women experience IPV. Women’s education, residence, sex of household head, current pregnancy, husband drinks alcohol, attitude towards wife beat, husband controlling behavior, and women’s decision-making autonomy were the major determinants of IPV. Therefore, we recommend establishing effective health and legal response services to IPV, raising awareness on the existing legislation service and improving its application, strengthening legislations on purchasing and selling of alcohol, strengthening joint (both husband and wife) decision making power by empowering women, improving the educational level of the women and establishing measures to break the culture of societal tolerance towards IPV.

The odds of women experiencing IPV were higher among women who had decision-making autonomy to visit their families. This may indicate a power struggle within the household. Women may be more vulnerable to IPV if women are empowered in the home without the support of men.

## Supplementary Information

**Additional file 1**. Questionnaire on violence against women.

## Data Availability

All data generated or analysed during this study are included in this manuscript.

## Abbreviations

AOR: Adjusted odds ratio
CI: Confidence interval
EA: Enumeration area
DHS: Demographic and Health Survey
ICC: Intra-cluster Correlation Coefficient
IPV: Intimate partner violence
LR: Likelihood ratio
