# Supplementary figures and images for: Magnitude and determinants of intimate partner violence against women in East Africa: multilevel analysis of recent demographic and health survey
Source: BMC Womens Health. 2022 Mar 17;22:74. doi: 10.1186/s12905-022-01656-7 (PMC8928594; doi:10.1186/s12905-022-01656-7)

**Appendix**


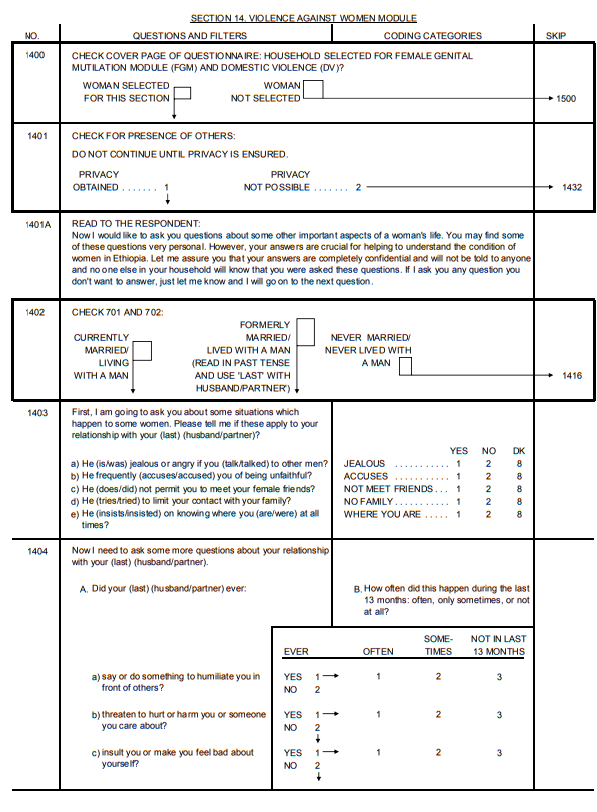


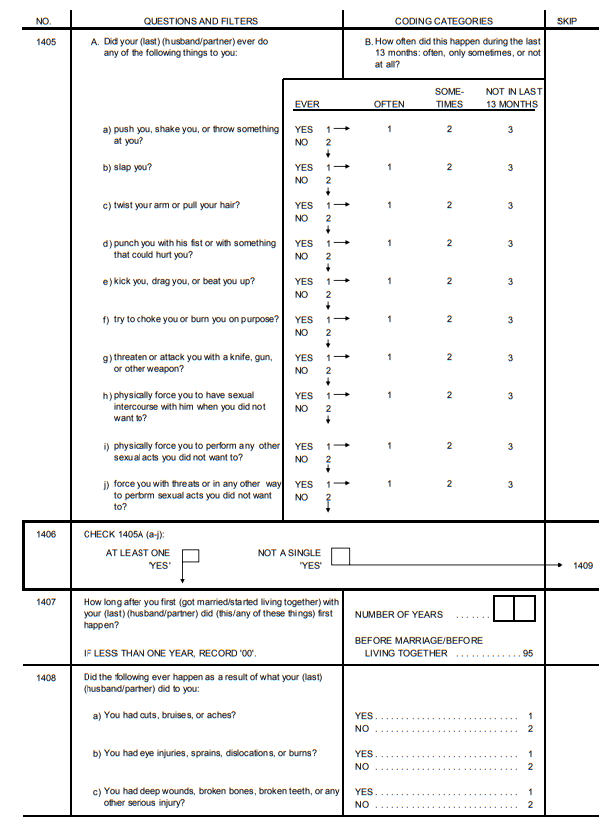


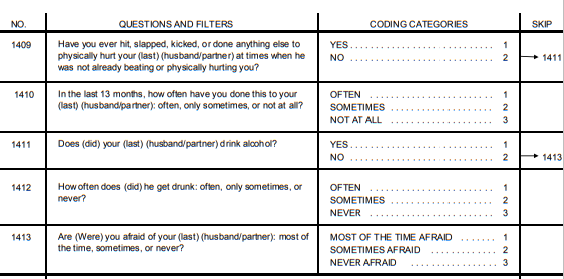

Supplement: Supplementary file 1 — Additional file 1. Questionnaire on violence against women. [file 12905_2022_1656_MOESM1_ESM.docx]
